# Sugar-sweetened beverage consumption and bone health: a systematic review and meta-analysis

**DOI:** 10.1186/s12937-021-00698-1

**Published:** 2021-05-05

**Authors:** Hyejin Ahn, Yoo Kyoung Park

**Affiliations:** 1 Department of Gerontology (AgeTech-Service Convergence Major), Kyung Hee University, Yongin, Republic of Korea; 2 Department of Medical Nutrition (AgeTech-Service Convergence Major), Graduate School of East-West Medical Science, Kyung Hee University, Giheung-gu, Yongin-si, Gyeonggi-do 17104 Republic of Korea; 3 Research Institute of Medical Nutrition, Kyung Hee University, Seoul, Republic of Korea

**Keywords:** Meta-analysis, Systematic review, Sugar-sweetened beverages, Carbonated beverages, Bone health, Bone mineral density, Bone fractures

## Abstract

**Background:**

Current evidence demonstrate that sugar-sweetened beverages (SSBs) and bone health are related; however, there has been only a few reviews on the link between SSBs and bone health. A systematic review and meta-analysis was performed to investigate the association between SSBs consumption and bone health in chidren and adults.

**Methods:**

Relevant studies of SSBs and bone health published up to 15 March 2021 were searched using PubMed, the Web of Science, Cochrane Library, and a reference search. A random-effects meta-analysis was conducted to estimate the standardized mean difference (SMD). Subgroup analyses were performed to identify whether effects were modified by age, sex, measured skeletal sites, type of SSBs, and SSBs intake questionnaire.

**Results:**

Twenty-six publications including 124,691 participants were selected on the review. The results from this meta-analysis showed a significant inverse association between SSBs intake and bone mineral density (BMD) in adults (ES: -0.66, 95% CI: − 1.01, − 0.31, *n* = 4312). Eighteen of the 20 studies included in the qualitative-only review in children and adults supported the findings from the meta-analysis. When subgroup analysis was performed according to skeletal site, a large effect was found on whole body BMD (ES: -0.97, 95% CI: − 1.54, − 0.40). There was a moderate effect on BMD in females (ES: -0.50, 95% CI: − 0.87, − 0.13). There was a moderate or large effect on BMD in individuals aged under 50 years (under 30 years: ES: -0.57, 95% CI: − 0.97, − 0.17; 30 to 50 years: ES: -1.33, 95% CI: − 1.72, − 0.93). High consumption of carbonated beverages had a moderate effect on BMD (ES: -0.73, 95% CI: − 1.12, − 0.35).

**Conclusion:**

The meta-analysis showed that SSBs consumption such as carbonated beverages were inversely related to BMD in adults. Qualitative review supported the results of meta-analysis.

**Trial registration:**

This review was registered in the PROSPERO database under identifier CRD42020164428.

**Supplementary Information:**

The online version contains supplementary material available at 10.1186/s12937-021-00698-1.

## Background

Sugar-sweetened beverages (SSBs), defined as any consumable non-alcoholic water-based beverage containing significant amounts of free sugars [1], are a primary source of sugar consumption [2], and the proportion of people consuming beverages as their major source of sugar is steadily increasing [2, 3]. This increase in sugar intake through beverages and its potential adverse effects on public health are of major concern [4]. SSBs include non-diet soft drinks/sodas; flavored juice drinks; sports drinks; sweetened waters; coffee, tea, and milk with added sugars; energy drinks; and electrolyte replacement drinks [5]. Strong evidence that SSB consumption is causally associated with increased risk of developing health problems, such as weight gain and obesity, type 2 Diabetes Mellitus, tooth decay, and cardiovascular disease, has been reported [1]. Accordingly, many research and policy efforts have focused on consumption of sugar-sweetened beverages due to their substantial contribution to total added sugar intake [6, 7]. The 2020 strategic Plan from the American Heart Association (AHA) recommends no more than 360 kcal per week from SSBs [8]. This recommendation is exceeded by over 80% of the population in the United States [8].

Bone metabolism is affected by a variety of environmental factors, especially dietary factors [9]. Given the increase in SSBs consumption over the past decade, many studies have been conducted to investigate the effect of SSBs consumption on bone health [10, 11]. Added sugar, phosphoric acid, caffeine, and the acidity of SSBs may all affect bone metabolism by disturbing calcium absorption and homeostasis in the body and increasing calcium excretion through urine [12–14]. High consumption of SSBs may also affect bone metabolism when replacing milk, known to be beneficial to bone health [15]. Also over consumption of SSBs is likely to accompany low diet quality (e.g. excessive intake of fast-food and low vegetable consumption), which might consequently influence micronutrient and calcium intake [16].

To date only one systematic review has shown a relationship between SSBs and childhood fractures [17]. However, this study focused on the relationship between calcium intake and bone fracture in children, so the association between SSBs and bone fractures was not considered important. Except for this study, there are no reviews on the relationship between SSBs and bone health.

We performed a comprehensive review of the literature as well as a meta-analysis to determine the association between SSBs consumption and bone health in children and adults.

## Materials and methods

The protocol for the systematic review and meta-analysis was registered a priori with the PROSPERO International Prospective Register of Systematic Reviews (CRD42020164428). PRISMA (Preferred Reporting Items for Systematic Reviews and Meta-analyses) guidelines were followed and the checklist was completed (Table S1).

### Data sources and searches

**Fig. 1:**
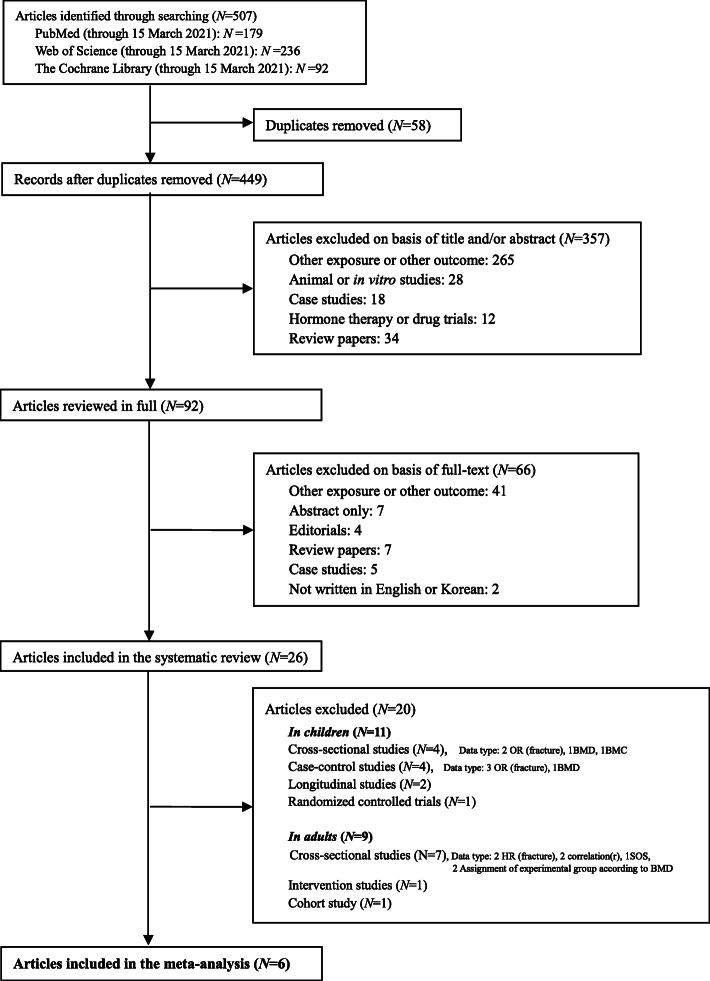
Flowchart of literature search and selection of studies

Systematic searches of PubMed, the Web of Science, and Cochrane Library databases for eligible studies published until the end of 15 March 2021 were performed. The search strategy is detailed in Table S2 in the Supporting Information online. Studies that investigated the association between SSBs consumption and bone health written in English and Korean were included. If the title, abstract, and keywords seemed relevant, the full-text of the record was assessed. Abstract, reviews and meta-analyses, studies with no relevant data, studies with non-relevant exposure or non-relevant outcomes, and duplicates were excluded. In addition, we performed manual searches of reference lists of relevant reviews and articles included in the systematic review. The literature search was performed without restrictions on study design or publication date. Ethical approval was not required for the present study. Further details of the search strategy are provided in Fig. 1.

### Study selection

The following criteria were applied to identify articles for inclusion in our systematic review and meta-analysis: 1) studies involving healthy people without coexisting medical conditions or treatments affecting bone metabolism; 2) studies presenting a relationship between SSBs consumption and bone-related parameters [bone mineral content (BMC), bone mineral density (BMD), or bone fractures]; and 3) studies reporting levels of bone-related parameters for groups with different consumption of SSBs. No study design or time restrictions were applied when searching the databases. Among the studies included in the systematic review, the criteria applied to the studies in the meta-analysis are as follows: 1) comparative studies of groups who consumed more than 1cup or less of SSBs per day; 2) studies presenting a relationship between SSBs consumption and BMD; 3) cross-sectional studies. We excluded studies with no comparative group, reviews, editorials, and in vivo*/vitro* studies.

Two investigators (H.A. and YKP.) independently screened the titles, abstracts, and keywords of the articles to evaluate eligibility for inclusion. If consensus was reached, articles were either excluded or a full text of the study was retrieved. Full texts of the selected articles were appraised critically to determine eligibility for inclusion in the review. Disagreements were resolved by discussion between the investigators until consensus was reached.

### Data extraction and quality assessment

All data were extracted by HA and checked by YKP. Extracted data included: 1) publication characteristics (author, publication year, geographic location, and sample size); 2) population (total number of participants, health status, age, sex ratio); 3) study design (intervention study, case-control study, or cross-sectional study); 4) exposure (assessment method and type of SSBs intake); 5) outcome (assessment method and bone-related parameters); 6) confounders (factors that analyses were adjusted for or matched on). If bone-related parameters were reported in different units to the most commonly used units, data were converted. When necessary, we contacted the authors of the primary studies to obtain additional information. Means and SDs were calculated for BMD or BMC and risk estimates were expressed as ORs, RRs or HRs with corresponding 95% CIs.

Two investigators (H.A. and Y.K.P.) independently evaluated the quality of cohort studies and case-control studies using the Newcastle–Ottawa quality assessment scale [18, 19] for the following criteria: representativeness and selection; comparability; assessment of outcome or exposure, which ranges 0 ~ 9 (good quality for ≥7, fair quality for ≥5). The quality of the intervention study was assessed using validated checklists published by the National Institutes of Health [20] ranging 0 ~ 14 (good quality for ≥11, fair quality for ≥8). The quality of cross-sectional (observational) studies was evaluated using Handel’s scale [17] for the reporting of observational studies in the field of nutrition. This quality assessment includes five quality categories: selection of study participants, measurement of outcome variables, description of withdrawals or dropouts, control for confounding bias, and application of an adequate statistic. Ten questions were given a grade based on a 5-point scale, and studies with scores of more than half of the total score were evaluated for good quality and fairness.

### Statistical analysis

BMD is usually expressed in g/cm^2^, and rarely as *Z* scores or *T* scores. To allow pooling of data, BMD values from each study were converted into a treatment effect size (ES) with its 95% CI, in accordance with the Hedges method, which is designed for quantitative data [21].

Statistical analyses were performed using the R program (mate-package, version 3.6.3). We conducted a meta-analysis according to a random-effects model (Hedge’s method) for the main effect outcomes by combining inverse variance-weighted study-specific estimates [22]. We calculated standardized mean difference (SMD) between two groups for measuring ES, which is used as a summary statistic in most of the meta-analysis when the studies all uses similar outcome measures, but, measured with various methods. Forest plots were used to visualize individual and summarize estimates, and the Cochrane Q statistic and *I*^2^ statistic calculated using the formula [(Q-df)/Q]× 100% were used to evaluate between-study heterogeneity [23, 24]. We considered an effect size of 0.30 or less to be “small”, an effect size of 0.40 to 0.70 to be “medium”, and an effect size of 0.80 or above to be “large” [25]. An *I*^2^ value > 50% was generally considered to be high [26]. Subgroup analysis to explore heterogeneity was performed with pre-specified potential confounders such as age, sex, measured skeletal sites, type of SSBs, and SSBs intake questionnaire [27, 28].

## Results

### Literature search and selection

We screened 507 references and confirmed 449 references as potential studies for review after removal of duplicates. Articles were excluded on the basis of title and/or abstract (*n* = 357), and then excluded on the basis of full text (*n* = 66). Finally, we included 26 references [29–54] involving 124,691 participants in this systematic review (*n* = 20) and meta-analysis (*n* = 6). Twenty studies were excluded from our meta-analysis, as the number of studies in which the study design and bone-related indicators matched was small. Excluded studies were reviewed qualitatively. Finally, the association between SSBs and BMD was assessed based on six references involving 4312 participants. The list of excluded reasons for exclusion are given in Fig. 1.

### Study characteristics

The characteristics of studies included in this systematic review and meta-analysis are summarized in Tables 1 and 2. Original articles in this review were published over the period from 1997 to 2020. The 26 studies included in the review were from the United States of America (*n* = 8), South Korea (*n* = 4), Egypt (*n* = 1), England (*n* = 2), Saudi Arabia (*n* = 2), Australia (*n* = 1), Canada (*n* = 1), Chile (*n* = 1), China (*n* = 1), Denmark (*n* = 1), Germany (*n* = 1), Greece (*n* = 1), New zealand (*n* = 1) and Norway (*n* = 1), with individuals aged from 4 to 98 years. The number of participants in these 26 studies totaled 124,691 and ranged from 98 to 73,572.

**Table 1:** Characteristics of the eight observational studies on associations between sugar-sweetened beverage consumption and bone health in children^a,b^

First author years (Ref) location	Study design	Sample size	Age or age range (Mean age ± SD)	Sex, % F	Sugar-sweetened beverages			Bone health			Main finding
					Method of *assessment*	*Beverage category*	Intake level	Method of assessment	Sites	Outcomes	
Albala 2008 [29] ^c^ Chile	Randomized controlled trial	98	8–10 y	46.9	Modified FFQ	Sugar-sweetened beverages^d^	Low: 742.8 ± 207.9^e^ High: 802.1 ± 142.0, *p* = 0.10 g/d	DEXA	WB	Bone Mass	∙No difference in whole body bone mass between children fed different amounts of sugar-sweetened beverages (*p* = 0.56).
Fisher 2004 [30] ^c^ USA	Longitudinal study	182	9 y	100	24-h dietary recall	Sweetened beverages^f^	Low: 358 High: 403, g/d	DEXA	WB	BMD	∙Girls who drank more sweetened beverages (*p* < 0.01) had a significantly lower whole body BMD (*p* < 0.001).
Libuda 2008 [31] Germany	Longitudinal study	228	6–18 y	49.6	3-day food records	Soft drinks	*8y, Prepubescent* Girls: 119.8 ± 129.2 Boys: 136.8 ± 137.3 *13y, Pubescent* Girls: 186.0 ± 196.5 Boys: 243.5 ± 200.4, g/d	pQCT	Forearm	BMC	∙Soft drinks consumption in children and adolescents was inversely associated with BMC at forearm (*p* = 0.036).
Ma 2004 [32] Australia	Case-control study	390	9–16 y	–	Questionnaire developed by author	Carbonated or cola drinks	Not reported	DEXA	WB LS FN	BMD	∙No significant correlation was shown between carbonated and/or cola drinks and bone measures, although all were inverse trends.
Manias 2006 [33] England	Case-control study	100	4–16 y	50	FFQ	Carbonated beverages	Low: 0.13 ± 0.17 High: 0.33 ± 0.57, *p* = 0.0182, ℓ/d	DEXA	LS UB LB	BMD BMC	∙Children who consumed more carbonated drinks (*p* = 0.0182) had a significantly lower BMD and BMC z-score at spine (BMD, *p* = 0.0003; BMC, *p* = 0.001), upper body (BMD, *p* = 0.015; BMC, *p* < 0.0001) and lower body (BMD, *p* = 0.015; BMC, *p* = 0.001).
McGartland 2003 [34] England	Cross-sectional study	1335	12–15 y	55.7	Dietary history	Carbonated soft drinks^g^	*12y*: Girls: 351 ± 332 Boys: 459 ± 394, *p* < 0.01 *15y*: Girls: 340 ± 380 Boys: 518 ± 452, *p* < 0.01, g/d	DEXA	DR HL	BMD	∙A significant inverse relationship between total intake of carbonated soft drinks and BMD was observed in girls at the forearm (*p* < 0.05) and heel (*p* < 0.05).
Nassar 2014 [35] Eqypt	Case-control study	100	Low: 10.3 ± 1.4y High: 10.6 ± 1.3y	44.1	Questionnaire developed by author	Sugar-sweetened beverages^d^	Low: 1.08 ± 0.64 *High*: 3.16 ± 0.37, *p* < 0.001, number of intake /day^h^	DEXA	LS	BMD	∙Children who consumed more than 12 oz had a significantly lower BMD (*p* < 0.001) than those that does not exceed 0–8 oz.
Whiting 2001 [36] Canada	Cross-sectional study	112	10–16 y	47.3	24-h recall	Carbonated and low nutrient-density beverages^i^	*Girls* Carbonated, 96 ± 102 Low nutrient dense, 240 ± 177 *Boys* Carbonated, 246 ± 300 Low nutrient density, 429 ± 393, mL/d	DEXA	WB	BMC	∙Consumption of carbonated (*p* = 0.05) and low nutrient dense beverages (*p* = 0.03) was inversely related to BMC in adolescent girls but not in boys.

^a^*BMC* bone mineral content, *BMD* bone mineral density, *d* day, *DEXA* Dual-energy X-ray absorptiometry, *DR* Distal radius, *F* Female, *FFQ* Food frequency questionnaire, *FN* Femoral neck, *High* high intake of SSBs, *HL* Heel, *LB* Lower body, *Low* low intake of SSBs, *LS* Lumbar spine, *pQCT* peripheral quantitative computed tomography, *QUS* Quantitative ultrasonography, *Ref* Reference, *SD* Standard deviation, *SOS* speed of sound, *WB* Whole body, *UB* Upper body, *USA* United States of America; y, year

^b^Quality assessment was performed using the Cochrane criteria and Handel’s-developed scale and assessed by two authors (HA and YKP)

^c^Standard errors (SE) presented in the original articles were converted to standard deviations (SD) for meta-analysis. This formula was used for conversion: SD=SEX√n

^d'^*Sugar-sweetened beverage*’ included carbonated beverages and juice drinks-made by adding packaged sugary powders with fruit flavoring to water

^e^Values are means ± SD (all such values)

^f^ ‘*Sweetened Beverage*’ included both energy-containing carbonated (soda) and noncarbonated beverages (fruit drinks, sport drinks, sweetened iced tea) that contained little if any fruit juice

^g'^*Carbonated soft drinks*’ were defined as all nonalcoholic carbonated beverages that contained artificial sweeteners instead of added sugar

^h^ First, children with a daily intake of more than 12 oz of SSBs or less than 0–8 oz of SSBs were recruited. After that, the number of drinks they consumed per day was investigated

^i'^*Carbonated beverages*’ includes cola, diet cola and other soft drinks; and ‘*low nutrient dense beverages*’ is the sum of carbonated and noncarbonated beverages. The latter included sugared drinks such as iced tea, Koolaid, coffee< 50% fruit juice, and fruit punches

**Table 2:** Characteristics of the twelve observational studies on associations between sugar-sweetened beverages consumption and bone health in adults^a,b^

First author, years (Ref), location	Study design	Sample size	Age range	Sex, % F	Sugar-sweetened beverage consumption			Bone health			Main finding
					Method of *assessment*	*Types*	Intake levels or categories	Method of assessment	Sites	Outcomes	
Alghadir,2015 [37] Saudi Arabia											∙Men and women in both younger and older groups who consumed more than 3 cups of carbonated beverages per day had a significantly lower whole body BMD than did those who consumed less than 3 cups (young, M, *p* < 0.01; young, W, *p* < 0.01; old, M, *p* < 0.01; old, W, *p* < 0.01)
Young, M Young, W	Cross-sectional study	100 86	25–30 y	46	Questionnaire developed by author^c^	Carbonated Beverage	Low: Normal^d,e^ (< 3 cups/wk) High: High (≥3 cups/wk)	DEXA	WB	BMD	
Old, M Old, W		60 104	31–45 y	63							
Cho 2008 [38] South Korea	Cross-sectional study	229	18–29 y	100	Questionnaire developed by author^f^	Carbonated Beverage	Low: Not at all High: Often (≥1 serving per day)	USM	HL	BMD SOS	∙No difference in heel BMD T-score between women who often consumed carbonated beverages and who did not consume carbonated beverages (*p* = 0.07). However, SOS levels of women who often consumed carbonated beverages was lower than those of women who did not consume carbonated beverages (*p* = 0.03).
Hammad 2017 [39] Saudi Arabia	Cross-sectional study	101	20–24.9 y	100	Modified FFQ^g^	Soft drinks	Low: Rare (< 1 can/d) High: Frequent (> 3 cans/d)	QUS	HL	BMD SI	∙Participants with frequent consumption of soft drinks had significantly lower T-scores and Z-scores for heel BMD than those with rare soft drink intake (Z-score, *p* = 0.02; T-score, *p* = 0.02). ∙Soft drink intake was inversely associated with T-score and Z-score of BMD and SI at the heel (T-score, *p* = 0.003; Z-score, *p* = 0.002; SI, *p* = 0.02).
Hostmark 2011 [40] Norway	Cross-sectional study	2126	30–60 y	59	FFQ	Soft drinks	Not reported	SEXA	DR	BMD	∙Cola and non-cola soft drink consumption was inversely associated with distal forearm BMD (cola, *p* = 0.012; non-cola soft drinks, *p* = 0.026), whereas consumption of fruit juice was not associated with distal forearm BMD when covariates were adjusted for.
Jeong 2010 [41] South Korea	Cross-sectional study	160	about 20	100	Modified FFQ^h^	Carbonated Beverage	Low: 51.3 ± 74.6 g/d High: 92.9 ± 114.1 g/d *p* < 0.05	USM	HL	BMD BUA SI	∙Women who consumed more carbonated beverages (*p* < 0.05) had significantly lower T-score (*p* < 0.001) and Z-score (*p* < 0.001) heel BMD, and showed significantly lower SI (*p* < 0.001) and BUA (*p* < 0.001).
Kim 1997 [42]^i^ USA	Cross-sectional study	1000	44–98 y	100	Questionnaire developed by author	Carbonated Beverage	Low: Nondrinkers or occasional drinkers High: Drinkers (≥1 serving per day)	DEXA	LS, TH DR, MR	BMD	∙No difference in BMD at the distal radius, mid-shaft radius, total hip, or lumbar spine was observed between women who drank or did not drink/ occasionally drank carbonated beverages.
Kim 2020 [43] South Korea	Cross-sectional study	2499	12–25 y	51	Dietary records	Cola	Low: Non-cola drinker High: Cola drinker	DEXA	WB, WF FN, LS	BMD	∙No difference in BMD at the whole body, whole femur, femoral neck, and lumbar spine was observed between participants who drank or did not drink carbonated beverage.
Kristensen 2005 [44] Denmark	Intervention study	11	22–29 y	0	–	Cola	2.5 L/d, during 10 days	–	Serum	OC, ALP, CTx, NTx	∙High consumption of cola over a 10-day period with a low-calcium diet reduced serum levels of OC. High intake of cola increased bone turnover compared to high intake of milk.
Pettinato 2006 [45] USA	Cross-sectional study	151	11–26 y	53	Modified FFQ	Soda	Girls: 2.1 ± 3.1 Boys: 1.1 ± 1.5, *p* = 0.012, cups/d	QUS	DR	SOS	∙Inverse correlation found between non-diet soda and the radical SOS at forearm in girls (*p* = 0.002), but not in boys.
Supplee 2011 [46] USA	Cross-sectional study	438	18 y<	100	FFQ	Soda	1.7 servings/d	QUS	HL	BMD	∙Soda consumption in the unadjusted model was positively and significantly associated with BMD (*p* < 0.0001). In the fully-adjusted model, however, soda consumption was no longer associated with BMD.
Tucker 2006 [47] USA	Cross-sectional study	2538	30–87 y	56	FFQ	Soft drinks	Not reported	DEXA	TH, TC FN, WA	BMD	∙Soft drink intake was associated with significantly lower BMD at each hip site (TH, *p* < 0.01; TC, *p* < 0.01; FN, *p* < 0.001; WA, *p* < 0.001) in women but not in men.
Yeon 2009 [48] South Korea	Cross-sectional study	133	18–23 y	100	Dietary records	Coffee with syrup or sugar	Low: 95.8 ± 163.5 g/d High: 194.5 ± 168.6 g/d, *p* < 0.05	USM	HL	BMD	∙No difference was observed in heel BMD between groups that consumed different amounts of beverages and coffee with sugar/syrup.

^a^*ALP* alkaline phosphatase, *BMD* bone mineral density, *BUA* broadband ultrasound attenuation, *CTx* c-terminal telopeptide, *d* day, *DEXA* dual-energy x-ray absorptiometry, *DR* distal radius, *F* female, *FFQ* food frequency questionnaire, *FN* femoral neck, *High* high intake of SSBs, *HL* heel, *Low* low intake of SSBs, *LS* lumbar spine, *MR* mid-shaft radius, *NTx* n-terminal telopeptide, *OC* osteocalcin, *QUS* quantitative ultrasound, *Ref* reference, *SD* standard deviation, *SEXA* single energy x-ray absorptiometry, *SI* stiffness index, *SOS* speed of sound, *WB* whole body, *WF* whole femur, *TC* trochanter, *TH* total hip, *USA* United States of America, *USM* ultra-sonometer, *WA* ward’s area, *wk.* week

^b^Quality assessment was performed using the Cochrane criteria and Handel’s-developed scale and assessed by two authors (HA and YKP)

^c^ Beverage consumption was subdivided into subcategories: i. Tea or coffee (caffeine-containing beverages), ii. Alcoholic beverages (alcohol, beer or wine), iii. Carbonated sugary beverages (such as cola beverages) or other soft drinks, iv. Milk intake

^d^Values are means ± SD (all such values)

^e^Participants with soft drink intake were divided into normal (less than average) and high groups (equal or more than average)

^f^The tool for lifestyle measurement consisted of 14 items known to be directly related to bone mineral density, including carbonated beverages

^g^A simple food-frequency questionnaire was used, indicating the number of times per week that these foods were eaten and whether the portion size was large in the case of soft drinks

^h^Based on Korean National Nutrition Survey 2008; frequently consumed food items based on amount and frequency were selected

^i^95% confidence interval (CI) presented in this original article was converted to standard deviation (SD) for meta-analysis using the following formula: SD = (Mean-Lower endpoint/1.96)X√n or SD = (Upper endpoint-Mean/1.96)X√n

Individual SSBs evaluated were carbonated/soda/soft beverages in 21 studies, sugar-sweetened beverages in four studies, and coffee with sugar or syrup in one study. The studies used a variety of methods to assess the amount of SSBs intake including food-frequency questionnaires (FFQs) or modified FFQs (*n* = 11), 24-h recall (*n* = 2), 3-day food record (*n* = 3), diet history (*n* = 1), and questionnaires developed by the authors (*n* = 8).

BMD and other bone-related parameters were measured by dual-energy X-ray absorptiometry (DEXA, *n* = 11), ultra-sonometry (USM, *n* = 3), quantitative ultrasonography (QUS, *n* = 3), peripheral quantitative computed tomography (pQCT, *n* = 1), or single energy x-ray absorptiometry (SEXA, *n* = 1) at the distal radius (DR), femoral neck (FN), forearm, heel, lower body (LB), lumbar spine (LS), mid-shaft radius (MR), whole body (WB), whole femur (WF), total hip (TH), trochanter (TC), and/or Ward’s area (WA).

### Qualitative review of studies not included in the meta-analysis

#### Association between SSBs consumption on bone health in children

The relationship between SSBs consumption and bone health in children and adolescents was investigated in eight articles (Table 1). Of these, six studies [30, 31, 33–36] reported a significantly inverse relationship between SSBs intake and bone health (two longitudinal studies, three cross sectional studies, and one case-control study). Two longitudinal studies [30, 31] performed over a 4-year period found a significant inverse relationship between SSBs consumption and forearm BMC (*p* = 0.036) or whole body BMD (*p* < 0.001) in children and adolescents. Three cross-sectional studies [33, 34, 36] also reported a significant inverse relation between SSBs intake and BMC (Manias: spine: *p* = 0.001; upper body, *p* < 0.0001; lower body, *p* = 0.001; Whiting: carbonated drinks, *p* = 0.05; low nutrient dense beverages, *p* = 0.03), BMD (Manias: spine: *p* = 0.0003; upper body, *p* = 0.015; lower body, *p* = 0.015; McGartland: forearm, *p* < 0.05; heel, *p* < 0.05), or speed of sound (SOS; forearm, *p* = 0.002). Of these, two studies in particular [34, 36] reported significant inverse relationships between SSBs consumption and bone health in girls only. One case-control study [35] presented inverse relation between SSBs consumption and BMD (*p* < 0.001) in children. The eight articles involving children and adolescents that studied the link between SSBs consumption and bone health had inconsistent study designs and bone-related parameters. Therefore, we could not conduct a meta-analysis of these studies.

#### Association between SSBs consumption on bone health in adults

The association between SSBs consumption and bone health in adults was investigated in twelve articles (Table 2). All six articles [40, 41, 44, 46, 47] excluded from the meta-analysis reported significant inverse associations between SSBs consumption and bone health in adults (five cross-sectional studies and one interventional study). Hostmark et al. [40] found a inverse association between SSBs intake and forearm BMD (cola, *p* = 0.012; non-cola soft drinks, *p* = 0.026). Supple et al. [46] reported an inverse association between SSBs consumption and heel BMD (*p* < 0.0001). Meanwhile, Tucker et al. [47] found a inverse association between SSBs intake and BMD in women (TH, *p* < 0.01; TC, *p* < 0.01; FN, *p* < 0.001; WA, *p* < 0.001) but not in men. Kristensen et al. [44] reported that SSBs consumption (cola 2.5 L/day) for 10 days increased bone turnover.

#### Association between SSBs consumption on bone fracture

Eight articles [32, 33, 49–54] examined the association between SSBs intake and bone fractures (Table 3). Three studies [49, 50, 52] reported the SSBs consumption was associated with an elevated HR or OR of bone fractures in adults (Chen: HR 4.69, 2.80, 7.88; Fung: RR 1.42, 1.15, 1.74; Kremer: HR 1.26, 1.01, 1.56). Four artivles [32, 50, 53, 54] found that excessive SSBs intake was associated with a higher bone fracture risk in children and adolescent (Delshed: Boy OR 2.0, 1.0, 4.3; Delshed: Girls OR 4.6, 2.3, 9.1; Ma: OR 1.39, 95% CI: 1.01, 1.91, *p* < 0.05; Petridou: cola OR 1.7, 95% CI: 1.2, 2.6, *p* = 0.007, non-carbonated beverages OR 1.6, 95% CI: 1.1, 2.3, *p* = 0.017; Wyshak: carbonated beverages OR 3.14, 95% CI: 1.45, 6.78, *p* = 0.004, colas OR 2.01, 95% CI: 1.17, 3.43, *p* = 0.011). The other case-control study [33] reported that SSBs intake was higher in the fracture group than the non-fracture group (fracture group: 0.25 ± 0.44 L/d; non-fracture group: 0.13 ± 0.17 L/d; *p* = 0.0161).

**Table 3:** Characteristics of the eight studies on the effect of sugar-sweetened beverage consumption on bone fractures in children and adults^a,b^

First author year (Ref) location	Study design	Sample size	Age range	Sex, % F	Main finding^c^
Chen 2020 [49] China	Cross-sectional and longitudinal study	9914	20–75 y	52	**Frequency of soft drinks consuption** ∙1-2times/wk.: HR 1.17 (0.81, 1.67) ∙3-4times/wk.: HR 1.13 (0.58, 2.21) ∙Almost Daily: HR 4.69 (2.80, 7.88) **Soft drinks consuption** ∙< 1 L/wk.: HR 0.96 (0.75, 1.24) ∙≥1 L/wk.: HR 1.16 (0.83, 1.61)
Delshad 2020 [50] New Zealand	Cross-sectional study	647	8–12 y	55	Boy OR 2.0 (1.0, 4.3)^d^ Girls OR 4.6 (2.3, 9.1)^d^
Fung 2014 [51] USA	Cohort study	73,572	50 y and older (postmenopausal women)	100	**Total soda** RR 1.42 (1.15, 1.74), *p* = 0.0004***^e^ RR 1.14 (1.06, 1.23) per daily serving^f^ **Regular soda** RR 1.37 (0.90, 2.10), *p* = 0.03* RR 1.19 (1.02, 1.38) per daily serving **Diet soda** RR 1.38 (1.06, 1.81), *p* = 0.007** RR 1.12 (1.03, 1.21) per daily serving **Caffeinated soda** RR 1.18 (0.82, 1.70), *p* = 0.02* RR 1.15 (1.02, 1.29) per daily serving **Non-caffeinated soda** RR 1.56 (1.16, 2.09), *p* = 0.19 RR 1.08 (0.97, 1.20) per daily serving **Cola** RR 1.18 (0.81, 1.71), *p* = 0.07 RR 1.12 (0.99, 1.26) per daily serving **Non-cola** RR 1.25 (0.87, 1.79), *p* = 0.007** RR 1.32 (1.08, 1.62) per daily serving
Kremer 2019 [52] USA	Cross-sectional and cohort study	27,617	50–79 y (postmenopausal women)	100	**Total soda** ∙Up to 2 serving/wk.: HR 1.03 (0.93, 1.13) ∙2.1–5 serving/wk.: HR 1.00 (0.88, 1.14) ∙5.1–14 serving/wk.: HR 1.07 (0.94, 1.23) ∙ > 14 serving/wk.: HR 1.26(1.01, 1.56)
Ma 2004 [32] Australia	Case-control study	390	9–16 y	–	**Cola drink** ∙Hand OR 1.41 (0.71, 2.82) ∙Wrist and forearm OR 1.39 (1.01, 1.91), *p* < 0.05* ∙Upper arm OR 0.65 (0.36, 1.17) **Carbonated drink** ∙Hand OR 1.11 (0.71, 1.74) ∙Wrist and forearm OR 1.14 (0.89, 1.46) ∙Upper arm OR 1.00 (0.63, 1.58)
Manias 2006 [33] England	Case-control study	100	4–16 y	50	∙SSBs intake(L/day) ∙Non-fracture groups: 0.13 ± 0.17 ∙Fracture group: 0.25 ± 0.44, p = 0.0161*^g^ -One fracture: 0.16 ± 0.19, *p* = 0.07163^g^ -Recurrent fractures: 0.33 ± 0.57, *p* = 0.0182*^f^, *p* = 0.0359*^h^
Petridou 1997 [53] Greece	Case-control study	200	7-14y	26	∙Carbonated non-cola beverages: OR 1.1 (0.7, 1.8), *p* = 0.641 ∙Cola beverages: OR 1.7 (1.2, 2.6), *p* = 0.007** ∙Non-carbonated beverages: OR 1.6 (1.1, 2.3), *p* = 0.017*
Wyshak 2000 [54] USA	Cross-sectional study	460	14-16y	100	∙Carbonated beverages: OR 3.14 (1.45, 6.78), *p* = 0.004** ∙Colas: OR 2.01 (1.17, 3.43), *p* = 0.011*

^a^*p < 0.05, **p < 0.01, ***p < 0.001. *95% CI* 95% confidence interval, *d* day, *F* female, *HR* Hazard ratio, *OR* odd ratio, *Ref* reference, *RR* risk ratio, *SD* standard deviation, *SSBs* sugar-sweetened beverages, *wk.* week, *y* year

^b^Quality assessment was performed using the Newcastle-Ottawa scale and Handel’s-developed scale and assessed by two authors (HA and YKP)

^c^Values are mean ± SD, ORs (95% CIs), RRs (95% CIs) or HRs(95% CIs)

^d^ORs for SSBs drinks and bone fractures when men and women who consumed ≥1 serving/d were compared with those consumed < 1 serving/d

^e^RRs for SSBs drinks and hip fractures when women who consumed ≥10 serving/wk. were compared with non-consumers

^f^RRs per serving per day (12 fluid ounces, 355 ml)

^g^*p* values refer to the significance of results compared to the non-fracture group (t-test)

^h^*P* values refer to the significance of results compared to the one fracture group (t-test)

### Quantitative review of associations between SSBs consumption and BMD in adults; *A meta-analysis*

**Fig. 2:**
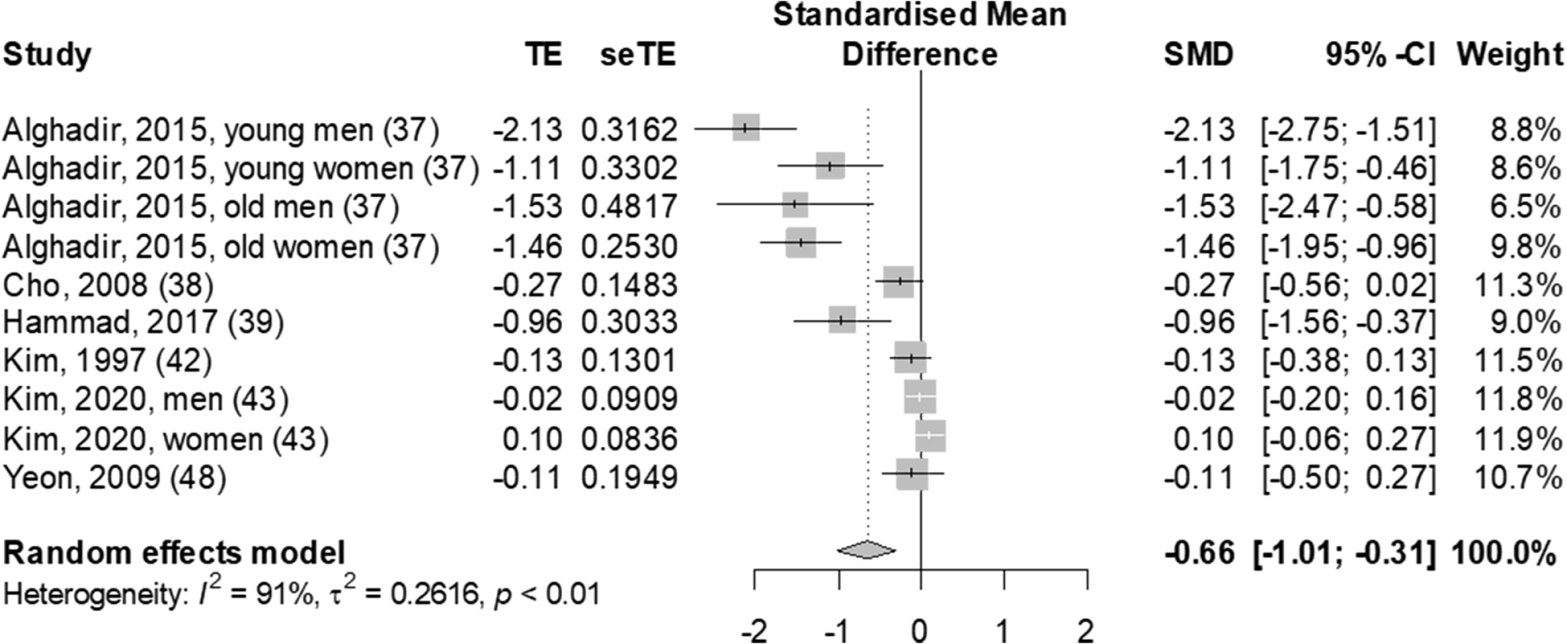
Forest plot of association between sugar-sweetened beverage consumption and bone mineral density in adults. SMD, standardized mean difference; 95% CI, 95% confidence interval; W weight. Numbers within brackets correspond to the citation number of the study. Squares and horizontal lines represent the effect size and 95% CI for individual studies, and the area of each square is proportional to the study's weight in the meta-analysis. Diamond and dashed vertical lines represent the overall effect size and 95% CI in the meta-analysis. The I^2^ and *P* values for heterogeneity are shown

The relationship between SSBs consumption and BMD in men and women is presented in Fig. 2, and individual effect sizes are shown in Table S3. This analysis was based on six studies including 4312 adults. We found a significant inverse association between SSBs consumption and BMD in adults (ES: -0.66, 95% CI: − 1.01, − 0.31; I^2^: 91%; quantifying heterogeneity test, *p* < 0.01). Among the six studies of adults, two studies [37, 39] found a significant inverse association between SSBs consumption and BMD. Meanwhile, the other four studies [42, 48] found no significant association. Alghadir et al. [37] reported a significantly lower whole body BMD in all four participant groups (young men, young women, older men, and older women) who drank ≥3 cups SSBs/week than in those who drank < 3 cups SSBs/week (SSBs, *p* < 0.01; BMD, *p* < 0.01). Hammad et al. [39] also reported that participants with frequent consumption of soft drinks (> 3 cans/day) showed significantly lower heel BMD T-scores and Z-scores that those with rare soft drink intake (< 1 can/day) (Z-score, *p* = 0.02; T-score, *p* = 0.02). Meanwhile, Cho et al. [38], Kim et al. [42] and Kim et al. [43] found no significant association between SSBs and BMD between participants who drank ≥1 serving/day and non−/occasional drinkers or between participants who drank SSBs ‘often’ or ‘not at all.’ Yeon et al. [48] also found no significant association between SSBs and BMD when comparing women who consumed an average of 194.5 g/day of SSBs and those who consumed an average of 95.8 g/day, even though the SSBs consumption of the two groups differed significantly (*p* < 0.05).

### *Qua*ntitative review of associations between SSBs consumption and BMD in adults; *A meta-analysis (Subgroup analysis)*

There was a significant inverse association between SSBs and BMD in adults (random-effects models, ES: -0.66, 95% CI: − 1.01, − 0.31) and significant heterogeneity (*I*^2^ = 91%; quantifying heterogeneity test, *p* < 0.01). We therefore conducted subgroup analyses according to age, sex, measured skeletal site and, type of SSBs, and SSBs intake questionnaire.

Individual effect sizes of subgroup analyses according to age, sex, measured skeletal site, type of SSBs, and SSBs intake questionnaire are summarized in Table 4. There was a large effect on whole body BMD (ES: -0.94, 95% CI: − 1.54, − 0.40). High consumption of SSBs had no effect on BMD at the heel, lumbar spine, total hip, mid-shaft radius, femoral neck, whole femur or distal radius. There was a moderate effect on BMD in females (ES: -0.50, 95% CI: − 0.87, − 0.13), but SSB consumption showed no association with BMD in males. There was a moderate or large effect on BMD in individuals aged under 30 years (ES: -0.57, 95% CI: − 0.97, − 0.17) and 30 to 50 years (ES: -1.33, 95% CI: − 1.72, − 0.93). Meanwhile, SSB consumption showed no association with BMD in participants older than 50 years of age (95% CI: − 0.34, 0.17). High consumption of carbonated beverages had a moderate effect on BMD (ES: -0.73, 95% CI: − 1.12, − 0.35), but consumption of coffee with sugar showed no association with BMD. Articles using the modified FFQ and questionnaire developed by author found a large effect on BMD (modified FFQ: ES -0.96, 95% CI: − 1.56, − 0.37; questionnaire developed by author: ES -1.05, 95% CI: − 1.68, − 0.42).

**Table 4:** Sub-group analysis of associations between sugar-sweetened beverage consumption and bone mineral density in different age groups, sex, skeletal site, SSBs type, and SSBs intake questionnaire^a^

	No. of studies	No. of participants	ES	95% CI	***I***^***2***^ (%)	***p***-value
***Age***						
**< 30 years**	**7**	**3122**	**-0.57**	**− 0.97 to − 0.17**^**b**^	**91**	***p*** **< 0.01**
**30–50 years**	**2**	**164**	**− 1.33**	**− 1.72 to − 0.93**^**b**^	**0**	**-**^**c**^
> 50 years	1	1000	− 0.13	− 0.38 to 0.13	-^c^	***p*** **= 0.40**
***Sex***						
Male	3	1382	−1.20	−2.75 to 0.36	96	*p* < 0.01
**Female**	**7**	**2930**	**− 0.50**	**−0.87 to − 0.13**^**b**^	**88**	***p*** **< 0.01**
***Skeletal site***						
Distal radius (g/cm^2^)	1	1000	0.06	−0.19 to 0.32	-^c^	-^c^
Femoral neck	2	2499	0.06	−0.11 to 0.24	53	*p* = 0.14
Heel (g/cm^2^)	3	463	−0.38	−1.78 to 0.01	65	*p* = 0.06
Lumbar spine (g/cm^2^)	3	3499	0.00	−0.19 to 0.20	68	*p* = 0.04
Mid-shaft radius (g/cm^2^)	1	1000	0.00	−0.26 to 0.26	-^c^	-^c^
Total hip (g/cm^2^)	1	1000	−0.19	−0.45 to 0.06	-^c^	-^c^
**Whole body (g/cm**^**2**^**)**	**6**	**2849**	**−0.97**	**−1.54 to − 0.40**^**b**^	**94**	***p*** **< 0.01**
Whole femur (g/cm^2^)	2	2499	0.03	−0.09 to 0.15	0	*p* = 0.40
***SSBs types***						
**Carbonated beverages**	**9**	**4179**	**−0.73**	**−1.12 to − 0.35**	**92**	***p*** **< 0.01**
Coffee with sugar	1	133	−0.11	−0.50 to 0.27	-^c^	-^c^
***Assessment method of SSBs***						
**Questionnaire developed by author**	**6**	**1579**	**−1.05**	**−1.68 to − 0.42**	**92**	***p*** **< 0.01**
24 h-dietary recall	3	2632	0.03	−0.08 to 0.15	0	*p* = 0.45
**Modified FFQ**	**1**	**101**	**−0.96**	**−1.56 to − 0.37**	-^c^	-^c^

^a^*95% CI* 95% confidence interval, *ES* effect size; *I*^2^, *FFQ* food-frequency questionnaires; I-square (%), *p p*-value, *SSBs* sugar-sweetened beverage. ES of 0.30 or less is regarded as small, 0.40 to 0.70 as medium, and 0.80 or above as large [24]. bEffect size is significant with 95% CI. ^c^Not applicable

## Discussion

In this systematic review and meta-analysis of 26 original articles, we identified an inverse association between consumption of SSBs and bone health. Meta-analysis of six studies revealed a significant inverse relationship between SSBs consumption and BMD in healthy adults. Eighteen of the 20 studies excluded from this meta-analysis supported its findings.

Sugar is thought to have negative effects on bone metabolism through increased loss of urinary calcium and imbalance in calcium homeostasis; thus the impact of excessive consumption of SSBs on bone health has become an area of intense research interest [11, 55]. We found that overconsumption of SSBs has an inverse relation on bone health as assessed by BMC, BMD, and the incidence of bone fractures. Carbonated drink or sugary coffee investigated in the relevant studies included in this review have other three major factors that influence bone metabolism beside sugar; phosphate, acidity, and caffeine [12–14]. Acids are added to beverages to provide a tart/tangy taste [56]. High phosphoric acid content affects calcium metabolism negatively, which when combined with low dietary calcium intake, could increase the risk of development of bone diseases [56, 57]. The low pH of carbonated drink such as cola (pH 1.8) can cause a sudden change in the gastric pH and thus interrupt calcium absorption, impairing bone health [56, 57]. Caffeine is another potential risk factor, although its role in bone loss is controversial [58]. Caffeinated beverage consumption, such as soda and sugary coffee, has been linked to reduced bone density and increased fracture rate [58]. High-fructose corn syrup (HFCS), or glucose-fructose syrup, is the main sweetener used in sugar-sweetened beverages [59]. Over-consumption of HFCS has been shown to be related to renal dysfunction and mineral imbalances, which could adversely affect bone health [60]. In a recent review paper, it was reported that caffeine consumption negatively affects the growth plate cartilage and bone health, through the alteration of pro-inflammatory and anti-inflammatory cytokines [61]. The main source of caffeine is soft drinks, coffee, tea, and chocolate. Another review paper showed that excessive dietary phosphorus intake have negative effects on bone metabolism [62]. This paper emphasized that soft drinks, in particular cola, is associated with altered bone metabolism, low bone density, and fracture in human studies [62].

Dietary calcium is the most important dietary factor for bone metabolism and bone health, and milk is an excellent source of calcium due to its high calcium content and high rate of absorption by the body [29, 44]. Apart from the direct effect of sugar itself, overconsumption of SSBs is strongly associated with reduced milk intake, resulting in lower bone mass and higher bone fracture risk through insufficient calcium intake [29, 44]. Eleven of the 22 original articles included in this review investigated relationships between SSBs and milk and/or intake of other calcium-containing products [30, 33–36, 41, 42, 45–48]. Of these, seven studies reported inverse correlations between SSBs and milk and/or calcium consumption [30, 33–36, 41, 47]. This review enabled us to confirm the relationship between high SSBs consumption and insufficient intake of milk and calcium. Two intervention studies that replaced milk with SSBs [29, 44] reported a link between milk and SSBs consumption. According to a short-term intervention study [44], high intake of cola combined with a low-calcium diet over a 10-day period induced increased bone turnover compared to high intake of milk with a low-calcium diet. Thus, the trend towards replacement of milk with cola and other soft drinks, which results in a low calcium intake, may inversely affect bone health. Another intervention study performed over 16 weeks [29] reported that replacing habitual consumption of SSBs with milk had beneficial effects on height, despite no changes in bone mass. Because milk and calcium intake are important for bone health [29, 44], overconsumption of SSBs, accompanied by a reduction in milk intake, inversely affects bone health.

Women tend to have smaller bones and lower bone strength as well as younger onset of bone loss than men, and are therefore particularly susceptible to osteoporosis [61, 62]. Subgroup analysis revealed that SSBs consumption had a significant inverse relation on BMD in women only. In addition, in four articles that investigated men and women separately, a significant inverse relationship between SSBs consumption and BMD or BMC was reported for girls and women, but not boys and men [34–36, 45, 47]. In two studies [51, 52] included in qualitative review, a positive association between SSB intake and bone fractures was confirmed in postmenopausal women. These findings suggest that excessive SSBs consumption is more detrimental to female bone health than male bone health.

Bone mass increases rapidly during childhood and adolescence, and up to 90% of peak bone mass accrues during this time [10]. Adolescence is known to be a period of remarkably high intake of SSBs, which is accompanied by a decrease in calcium and milk intake, and diet quality is often low [10, 11, 63]. We found that frequent consumption of SSBs in adolescence had a detrimental effect on bone health and was often associated with low calcium, milk, or protein consumption, which play an important role in bone health. These eating habits may make it difficult to acquire adequate bone mass or achieve peak bone mass, thereby increasing the risk of age-related osteoporosis in the future as well as increasing the risk of bone fractures in children. According to a longitudinal study [29], children who had a habit of overconsuming SSBs had low calcium, milk, vitamin D, and protein intake despite the importance of these factors in bone health. In addition, evidence is accumulating that suggests that eating habits in childhood have a great effect on eating habits as an adult [64]. Therefore, efforts to control children’s excessive intake of SSBs and to encourage healthy eating habits are important for maintaining healthy bone health and improving quality of life later in life.

Bone fractures, which are indicators of bone health, are also linked to SSBs consumption [32, 34]. Frequent intake of SSBs and failure to achieve bone mass can ultimately increase the risk of fracture [51, 53]. In all eight stidies included in this review, a positive association between SSB intake and bone fractures was confirmed in children and adults. In seven studies [32, 49–54], high consumption of carbonated beverages increased the risk of bone fracture by 1.3- to 4.69-fold. The one study [33] reported two-fold higher SSBs intake in participants with bone fractures and three-fold higher SSBs intake in participants with recurrent bone fractures compared to their counterparts. Our review confirms that overconsuming SSBs not only affects bone health, but also overall quality of life through increased bone fracture. Policy targets, such as those discussed in this report and summarized below, are needed to reduce sugary drink consumption in children and adolescents and subsequently improve child health. The relevant studies included in the meta-analysis set the standard for SSB intake to 1 ~ 3 servings per day or 3 servings per week. In participants who consumed more or less than this amount, SSBs consumption and BMD showed a significantly inverse relationship. The 2020 strategic Plan from AHA recommends no more than 3 ~ 4serving(360 kcal) per week from SSBs [8]. The results may suggest that observing this recommendation could help maintain bone health in addition to lowering the risk of obesity, diabetes and cardiovascular disease.

Many systematic reviews and meta-analyses related to SSBs consumption have been conducted [65, 66]. However, the majority of these reviews have focused on prevention of unhealthy weight gain/obesity [4] and associated conditions, such as type 2 diabetes, dental caries, or dyslipidemia [65, 66]. Meanwhile, there have been few reviews on bone health. This review provides useful information about the relationship between SSBs intake and bone health. This review is, to the best of our knowledge, the first systematic review and meta-analysis to evaluate the association between SSBs consumption and bone health in children and adults. Our findings indicate that SSBs consumption is inversely related to bone health in children and adults. According to the Centers for Disease Control and Prevention (CDC), SSBs consumption is higher among low-income families than high-income families [6, 67]. Therefore, policies and efforts to lower SSBs intake may improve the health of low-income individuals. There has been much discussion about the effects of SSBs on obesity and diabetes, and based on these evidences, limits on the intake of SSBs have been established. However, the effect of SSB on bone health has been inadequately addressed. It takes a very long time to determine the effects of dietary consumption on bone health. Therefore, research on this topic is bound to be limited. However, this topic is an area that must be studied for public health, and that this study will be the cornerstone.

However, some limitations of this review should be noted. First, most of the individual studies included in this review were cross-sectional observational studies. However, we were aware that the nature of this topic would limit inclusion of many other study designs Second, BMD in the included studies was not adjusted for potential confounders (gender, age, height, weight, physical activity, smoking, and alcohol use, among others). We addressed this limitation by conducting quality assessment, which included an evaluation category, to account for whether adequate adjustments had been made for confounding factors. Third, only six original articles were included in the meta-analysis; therefore, we were not able to evaluate heterogeneity in sub-group analyses. However, most of the 16 studies that were not included in the meta-analysis but included in the qualitative systematic review showed similar observations to those of the meta-analysis, gave strong supporting evidence to the results of the meta-analysis. Fourth, studies included in this review used different methodology of food intake investigation such as food records, 24-h dietary recall, FFQ, or dietary history. While all of which are proven to be valid as common methods in food intake survey research, the inconsistencies in the methods are inevitable in this kind of study. Fifth, it was difficult to analyze the publication bias, because the number of studies included in the analysis is small.

## Conclusion

In conclusion, this meta-analysis showed a significant inverse association between consumption of SSBs and BMD. The results of the qualitative review supported the finding that SSBs intake were linked to bone health. There has been a worldwide effort to reduce excessive consumption of SSBs by approaches including nutrition education, campaigns, and putting policies in place. We have confirmed that these efforts not only prevent obesity, diabetes, and cardiovascular disease, but also have a beneficial effect on public bone health.

## Supplementary Information

**Additional file 1: Table S1.** PRISMA 2009 checklist. **Table S2.** Search strategy for a systematic review and meta-analysis assessing the association between SSB consumption and bone health in children and adults. **Table S3.** Individual effect sizes from studies included in the meta-analysis.

## Data Availability

All data generated or analyzed during this study are included in this published article.

## Abbreviations

ALP: alkaline phosphatase
BMC: bone mineral content
BMD: bone mineral density
BUA: broadband ultrasound attenuation
CTx: c-terminal telopeptide
DEXA: dual-energy x-ray absorptiometry
DB: distal radius
FFQ: food-frequency questionnaire
FN: femoral neck
HL: heel
HR: hazard ratio
LB: lower body
LS: lumbar spine
MR: mid-shaft radius
NTx: n-terminal telopeptide
OC: osteocalcin
pQCT: peripheral quantitative computed tomography
QUS: quantitative ultrasonography
RCT: randomized controlled trials
SEXA: single energy x-ray absorptiometry
SI: stiffness index
SMD: standardized mean difference
SOS: speed of sound
SSBs: sugar-sweetened beverages
TC: trochanter
TH: total hip
UB: upper body
USM: ultra-sonometer
WA: ward’s area
WB: whole body
